# Hydroxyapatite Deposition Disease: A Comprehensive Review of Pathogenesis, Radiological Findings, and Treatment Strategies

**DOI:** 10.3390/diagnostics13162678

**Published:** 2023-08-15

**Authors:** Tarek Hegazi

**Affiliations:** Department of Radiology, College of Medicine, Imam Abdulrahman Bin Faisal University, Dammam 34212, Saudi Arabia; tmhejazi@iau.edu.sa

**Keywords:** hydroxyapatite deposition disease, HADD, etiology, radiological findings, differential diagnosis, radiologist role

## Abstract

Hydroxyapatite deposition disease (HADD) represents a multifaceted condition characterized by the accumulation of hydroxyapatite crystals in soft tissues, leading to subsequent inflammation and discomfort. The intricate etiology of HADD is the subject of this comprehensive review, which encompasses an in-depth analysis of the four proposed pathogenic mechanisms and a deliberation on the predisposing factors that instigate the development of this disease. In order to provide a thorough understanding of the disease’s progression, this manuscript delineates the stages of HADD—those preceding calcification, occurring during calcification, and following calcification—in meticulous detail. This chronology forms the basis of a complete portrayal of the evolution of HADD. Moreover, this review encompasses an examination of the radiological findings associated with HADD, furnishing an extensive discourse on imaging characteristics. The potential of HADD to mimic other diseases, thereby posing diagnostic challenges, is also articulated. The discourse continues with an investigation of HADD’s differential diagnosis. This section furnishes a robust framework for distinguishing HADD from other conditions based on imaging results. To enrich the understanding of this diagnostic process, case studies illustrating real-world applications are provided. An overview of treatment modalities for HADD, including both conservative and interventional approaches, forms the concluding discussion. The pivotal role of imaging specialists in the diagnosis and management of HADD is emphasized, highlighting their vital contribution to image-guided procedures and disease monitoring.

## 1. Introduction

Hydroxyapatite deposition disease (HADD), alternatively known as calcific tendinosis, calcific periarthritis, peritendinitis calcarea, calcific peritendinitis, calcific bursitis, and hydroxyapatite rheumatism, is a condition characterized by calcium hydroxyapatite crystal deposits in tendons, joints, and other soft tissues [1]. The disease is often asymptomatic and self-limiting, but when it is associated with an inflammatory process or when it occurs in unusual locations, it can imitate trauma, infection, or neoplasm [2], leading to misdiagnosis and unnecessary interventions [3]. It has a higher prevalence in females compared to males (2:1) [4].

Our understanding of HADD has significantly evolved over the years, with advancements in both diagnostic techniques and therapeutic approaches. Radiological imaging plays an instrumental role in the diagnosis and management of HADD. Despite the glenohumeral joint being the most common location for HADD [5], the diverse clinical presentations and locations of HADD present a considerable diagnostic and management challenge, underscoring the need for continuous research and review of the disease.

HADD’s etiology is not entirely understood, but it is theorized to involve a series of stages, encompassing a precalcific stage, a calcific stage, and a postcalcific stage [6]. The precalcific stage involves changes in the tendon cells that predispose them to calcification. The calcific stage is marked by the actual deposition of calcium hydroxyapatite crystals in the tendon [7]. The postcalcific stage involves the resolution of the calcific deposit and the healing of the tendon [8].

Comprehensive literature reviews bear vital importance in the realm of medical research. They synthesize a vast amount of research, facilitating a better understanding of the current state of knowledge, identifying research gaps, and setting directions for future studies. This comprehensive review of hydroxyapatite deposition disease aims to delve into the etiology, underscore the pivotal role of radiological findings in diagnosis, and explore the current and emerging treatment strategies for this condition.

## 2. Pathogenesis of HADD

### 2.1. Explanation of the Four Proposed Pathogenic Pathways

The precise etiology of hydroxyapatite deposition disease (HADD), a multifaceted condition, remains elusive and is an ongoing subject of investigation. The development of HADD, despite the current gap in definitive understanding, is potentially elucidated through four proposed primary pathogenic mechanisms [9] (Table 1).

#### 2.1.1. Degenerative Calcification

Degenerative calcification represents the first hypothesis in the potential development of HADD. According to this theory, the deposition of calcified material may be incited by localized damage to the tendon, attributable to either vascular ischemia or repetitive trauma [10].

Vascular ischemia describes a deficit in the blood supply, leading to insufficient oxygen and glucose required for cellular metabolism. For tendons, vascular ischemia could be a consequence of multiple factors such as physical injury, repetitive strain, or medical conditions that obstruct blood circulation [11]. The aftermath of vascular ischemia on a tendon potentially leads to tissue necrosis, initiating a series of biological responses including inflammation and chemical release, promoting calcification [11].

Similarly, repetitive trauma, or damage from recurring physical stress on the tendon, could stem from activities with repetitive motions like certain sports or jobs. The consequence of such trauma is often microtears in the tendon, leading to inflammation and subsequent tissue degeneration. This degenerated environment potentially facilitates calcium deposition, resulting in calcification [10].

This path of degenerative calcification indicates that HADD is a response to physical stress or injury involving tissue degeneration followed by calcification or the accumulation of calcium salts. While typically a part of the body’s healing process, in HADD, this results in hydroxyapatite crystal formation, which induces pain and inflammation [11].

#### 2.1.2. Reactive or Cell-Mediated Calcification

Reactive or cell-mediated calcification is the second theory, suggesting that chondrocytes, or the cells maintaining the cartilaginous matrix, mediate calcium deposition [12]. Chondrocytes, residing in cartilage, synthesize and maintain the cartilaginous matrix, a network of proteins and molecules that provide structure and support to the tissue. In the context of HADD, chondrocytes may contribute to calcium deposition, potentially in response to injury or inflammation [12]. In addition, this pathway involves phagocytosing cells, which absorb harmful foreign particles, bacteria, and dead or dying cells. These cells aid in the resorption of calcified material, implying that the body’s cells play a part in both HADD development and resolution [13].

#### 2.1.3. Process Similar to Endochondral Ossification

Endochondral ossification, a fundamental bodily process responsible for long bone formation and natural growth, is hypothesized to be similar to the third mechanism of HADD pathogenesis. In HADD, this process deviates pathologically, resulting in abnormal calcium deposition within the soft tissues rather than the standard transformation of cartilage into bone. This deviant calcification can occur in tendons, ligaments, and other periarticular soft tissues, leading to HADD’s clinical manifestations. The specific trigger for this abnormal ossification remains elusive, but it is conjectured to be a response to tissue damage or stress, similar to the physiological injury response where bone forms to repair and strengthen the damaged area [14].

#### 2.1.4. Erroneous Differentiation of Tendon-Derived Stem Cells

The last hypothesized mechanism involves the faulty differentiation of tendon-derived stem cells into calcium-depositing chondrocytes or osteoblasts. Stem cells, particularly tendon-derived ones, hold the capacity for self-renewal and differentiation into various cell types, including tenocytes, chondrocytes, and osteoblasts [15].

In the context of HADD, these tendon-derived stem cells are hypothesized to mis-differentiate into calcium-depositing chondrocytes or osteoblasts, contributing to disease development. This implies a dysregulation in the stem cell differentiation process, possibly due to genetic or environmental factors. The mis-differentiated cells then contribute to the formation of hydroxyapatite crystals within soft tissues, leading to HADD’s clinical manifestations [15].

This pathogenic mechanism suggests that stem cells may play a part in HADD’s pathogenesis, pointing to a potential novel treatment approach. However, further investigation is required to fully comprehend this mechanism and to develop effective stem cell-based treatments for HADD [15].

In conclusion, the complexity of HADD’s pathogenesis likely involves multiple pathways, including processes similar to endochondral ossification and erroneous differentiation of tendon-derived stem cells. Comprehending these mechanisms is crucial for devising effective HADD treatments. Yet, further exploration is necessary to fully unravel these mechanisms and their treatment implications for HADD [15].

## 3. Predisposing Risk Factors for HADD

There are a number of predisposing factors that augment the likelihood of the onset of hydroxyapatite deposition disease (HADD) (Table 2). Metabolic and genetic influencers hint at a multifaceted etiology underpinning this disease [3]. Diabetes is a substantial risk factor associated with HADD. The exact contribution of diabetes to HADD is yet to be fully delineated. Nevertheless, diabetes-induced metabolic alterations, such as chronic low-grade inflammation and heightened oxidative stress, are speculated to foster the deposition of hydroxyapatite crystals within the soft tissues [16].

Another aspect to consider in the metabolic sphere are disorders pertaining to thyroid and estrogen metabolism, both of which have been associated with HADD. The thyroid gland’s pivotal role in regulating metabolism and the widespread physiological consequences of its dysfunction are of interest here [17]. Similarly, estrogen, largely implicated in female reproductive health and bone metabolism, can induce abnormal calcium metabolism if imbalanced, potentially resulting in hydroxyapatite crystal deposition in soft tissues [18].

Beyond these metabolic elements, certain genetic factors are also found to be linked with an increased HADD risk. The HLA-A1 genotype, part of the human leukocyte antigen (HLA) system that is critical to immune recognition of self and non-self cells, is one such genetic factor. The HLA-A1-HADD association hints at a potential immunological dimension to the disease, though the precise mechanism remains to be elucidated [19].

Considering these factors, the etiology of HADD is likely influenced by a complex interplay between metabolic and genetic components. These may interact with the proposed pathogenic pathways, potentially instigating the disease in susceptible individuals. Metabolic disorders like diabetes, and disorders of thyroid and estrogen metabolism, might induce cellular stress or damage, triggering the degenerative calcification pathway (16,17,18). Similarly, the HLA-A1 genotype might influence the reactive or cell-mediated calcification pathway, thus promoting hydroxyapatite crystal deposition [19].

To conclude, the emergence of HADD is thought to be influenced by the complex interplay between metabolic and genetic components. Unraveling these risk factors and their interaction with the proposed pathogenic pathways is imperative for devising efficacious preventive and therapeutic strategies for HADD. However, a comprehensive understanding of these interactions and their role in the pathogenesis of HADD demands further research [3].

## 4. Stages of HADD

### 4.1. Description of the Precalcific, Calcific, and Postcalcific Stages

The progression of hydroxyapatite deposition disease (HADD) is a temporal phenomenon that can be categorized into three primary stages: precalcific, calcific, and postcalcific [20] (Table 3).

The precalcific stage serves as the inception of HADD and is characterized by reduced perfusion and resultant localized hypoxia, triggering fibrocartilaginous transformation at the prospective calcification site. Often asymptomatic, this stage can be overlooked until the progression to the subsequent stage [12,21,22].

The calcific stage signifies the calcium crystal deposition phase and is divided into formative, resting, and resorptive phases. The formative phase sees fibrocartilage being replaced by a calcific deposit, which during the resting phase, resides without associated vascularity or inflammation for a variable duration. Eventually, the resorptive phase sees the calcium deposit being invaded by macrophages, polymorphonuclear cells, and fibroblasts that phagocytose and remove the calcium, giving the calcification an ill-defined, irregular appearance. The acute pain associated with HADD typically coincides with the resorptive phase, wherein the calcific deposit may rupture into nearby tissues, inciting an acute inflammatory response. Corresponding MRI observations report edema or a fluid signal surrounding the calcific focus [12,21,22].

The postcalcific stage is a critical juncture in HADD progression. At this stage, the body initiates the repair of calcification-induced damage. Granulation tissue, comprising new connective tissue and microscopic blood vessels, replaces the void left by the resorptive phase. This tissue matures into scar tissue over time, indicating the termination of the active disease phase. However, this does not always indicate a cessation of symptoms or treatment requirement, as the scar tissue can induce discomfort and restrict function depending on its location [12,21,22].

### 4.2. Explanation of the Changes in HADD over Time

The progression of HADD is a dynamic, individualized process, varying in duration and severity among individuals. Certain individuals may rapidly progress from the precalcific to the postcalcific stage, while others may stay in a single stage for an extended period with minimal disease alteration [4]. Notably, symptom severity does not necessarily align with the disease stage. The resorptive phase, despite being part of the calcific stage, is often the most acutely painful. This phase, involving the body’s attempt to breakdown and remove calcium deposits, can cause significant inflammation and pain. However, the initiation of the postcalcific stage typically coincides with pain resolution and the commencement of the healing process [8].

Understanding HADD’s progression, including the postcalcific stage, is critical for disease management. This knowledge can inform treatment decisions and provide patients with a better grasp of their condition. Despite the potential for significant pain and discomfort, it is crucial to remember that HADD is a self-limiting disease that typically resolves over time, particularly as the body enters and progresses through the postcalcific stage [8].

## 5. Radiological and Imaging Findings of HADD

Hydroxyapatite deposition disease (HADD), a condition characterized by the deposition of hydroxyapatite crystals, showcases a broad spectrum of radiological findings discernible in multiple imaging modalities. A comprehensive understanding of these findings aids in the accurate diagnosis of HADD, setting it apart from other conditions with analogous symptoms [23].

Hydroxyapatite crystals, the focal point of HADD, are predominantly radiopaque, translating to augmented density zones on radiographs. During the initial stages of HADD, these deposits appear scattered and minuscule, posing a challenge for detection. As the disease advances, these deposits merge into larger clusters, making them more visible [10].

### 5.1. Detailed Discussion on the Imaging Features of HADD

#### 5.1.1. Plain Radiographs

**Figure 1:**
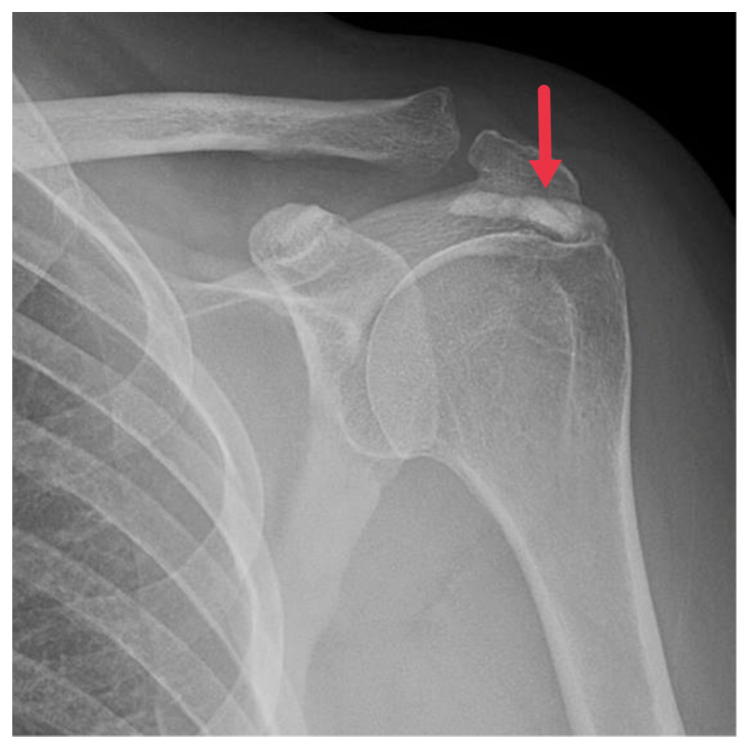
Frontal radiograph of the left shoulder demonstrating a well-defined calcification in the supraspinatus tendon (red arrow).

Regarded as the first line of imaging modality for HADD diagnosis, plain radiographs capture the hallmark feature of HADD: amorphous, nebulous densities in soft tissues. These hydroxyapatite densities mainly encircle joints, particularly the shoulder (Figure 1), but can also inhabit unconventional regions like the hip, wrist, and spine.

Advanced cases of HADD might exhibit these deposits in conjunction with bone erosion or cortical irregularities [3,8]. The scattered distribution of the calcified material accounts for the nebulous appearance of these deposits on radiographs, setting HADD apart from other conditions with similar radiographic findings [3,8,21]. The location of these deposits could provide valuable insights for diagnosing HADD. Instances of deposits in areas like the hip or wrist might point towards HADD in patients with discomfort in these areas. 

Although rare, deposits in the spine could be indicative of HADD [24]. Progressed HADD stages might show hydroxyapatite deposits accompanied by bone erosion or cortical irregularities. Bone erosion, the dissolution of bone tissue, might occur due to the pressure exerted by the hydroxyapatite deposits on the neighboring bone tissue. Cortical irregularities, anomalies in the bone’s outer layer, could be due to various factors, including HADD [24].

#### 5.1.2. Computed Tomography

**Figure 2:**
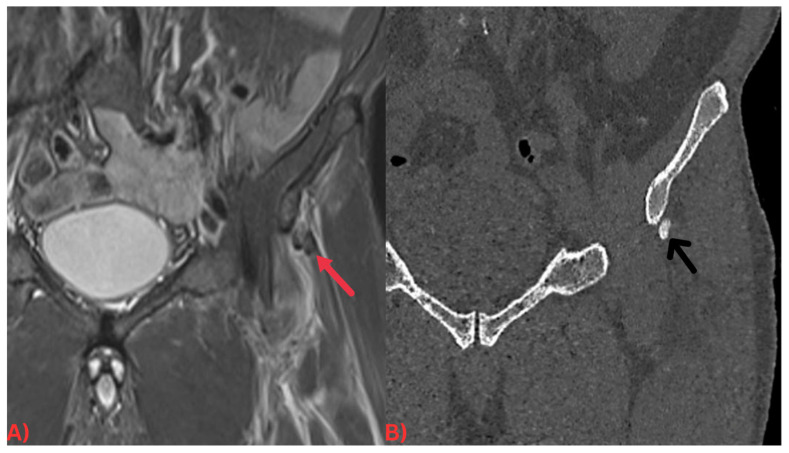
(**A**) Coronal short tau inversion recovery (STIR) MRI of the pelvis demonstrating an ill-defined low signal intensity focus at the origin of the left rectus femoris tendon (red arrow) with surrounding soft tissue edema. (**B**) Coronal CT image of the pelvis. Note how the calcification is more easily discernable on the CT image (black arrow).

Computed tomography (CT) offers an in-depth visualization of hydroxyapatite deposits, elucidating their size, shape, and location. CT scans reveal these deposits as high-density zones within soft tissues, often associated with the inflammation around them, apparent as increased attenuation in adjacent soft tissues [23] (Figure 2). The high-density portrayal of hydroxyapatite deposits on CT scans stems from hydroxyapatite’s high calcium content, which absorbs more X-rays than the surrounding soft tissues [23]. The detailed information offered by CT scans can shape treatment strategies for HADD patients, such as by guiding surgical interventions [25]. The hydroxyapatite deposits’ association with surrounding inflammation can be gauged on a CT as decreased attenuation in adjacent soft tissues from the soft tissue edema. This observation can confirm the diagnosis of HADD and evaluate the disease’s extent [25].

#### 5.1.3. Magnetic Resonance Imaging 

Magnetic resonance imaging (MRI) constitutes an essential imaging modality for diagnosing soft tissue modifications linked to hydroxyapatite deposition disease (HADD) [26]. Non-invasive in nature, MRI utilizes a robust magnetic field and radio waves to generate comprehensive images of the body’s internal structures, providing key insights into the hydroxyapatite deposits affiliated with HADD.

**Figure 3:**
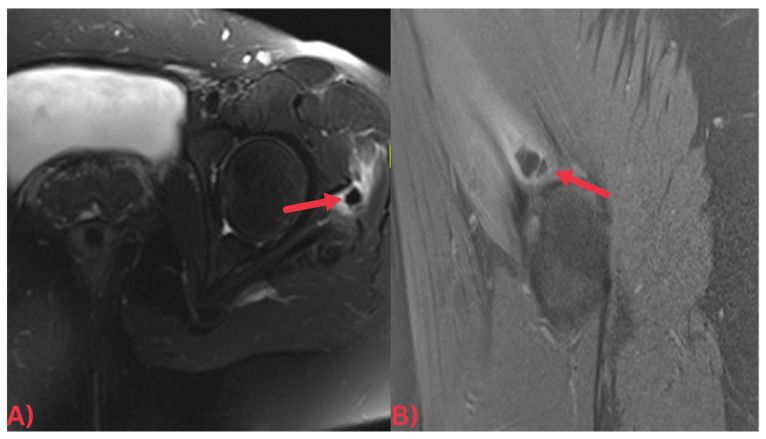
(**A**) Axial T2 fat-saturated MRI image and (**B**) sagittal PD fat-saturated MRI image of the left hip demonstrating a well-defined low signal intensity calcification at the insertion of the left gluteus medius tendon (red arrows) with surrounding soft tissue edema.

**Figure 4:**
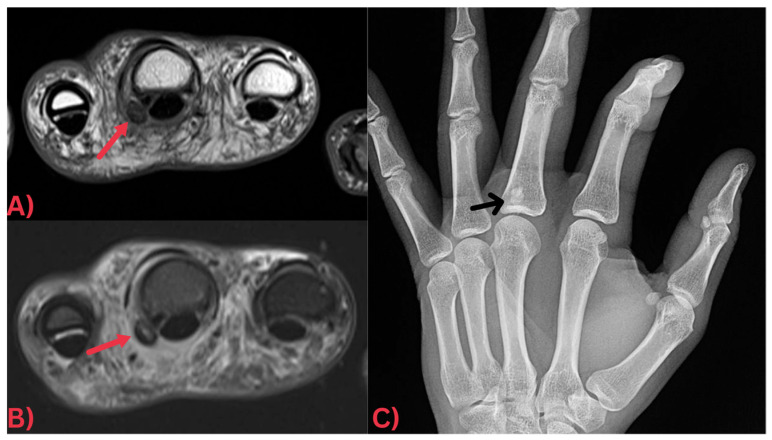
(**A**) Axial PD and (**B**) axial T2 fat-saturated MRI images of the hand demonstrating a well-defined globular low signal intensity focus at the volar capsule of the metacarpophalangeal joint of the middle finger (red arrows) with significant surrounding soft tissue edema. Note how this focus is more easily discernable on the (**C**) X-ray image of the hand, which clearly demonstrates that it is a focus of calcification (black arrow).

**Figure 5:**
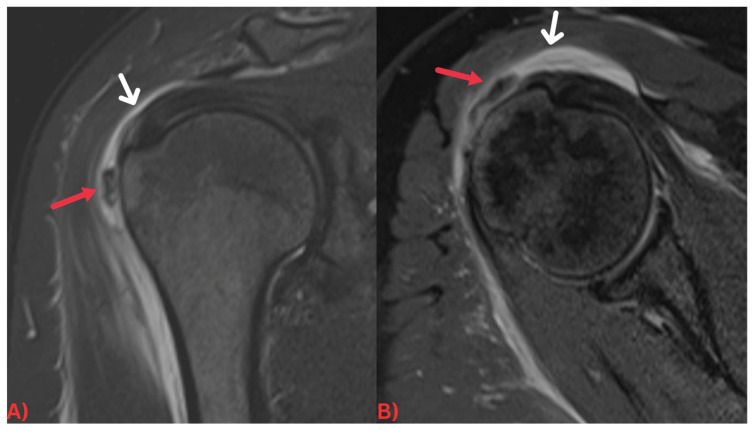
(**A**) Coronal PD fat-saturated MRI and (**B**) axial PD fat-saturated MRI images of the right shoulder demonstrating a few ill-defined low signal intensity calcifications (red arrows) in the subacromial/subdeltoid bursa, with fluid signal intensity within the bursa (white arrows) most suggestive of calcific bursitis.

Specifically, MRI images display hydroxyapatite deposits as regions with low signal intensity across both T1- and T2-weighted images, attributable to the high mineral content of hydroxyapatite, a form of calcium phosphate crystals [27]. Consequently, this results in its manifestation in MRI images (Figure 3). Concurrently, MRI also reveals inflammation signs within surrounding soft tissues, such as edema and increased signal intensity on T2-weighted images [28] (Figure 4). Edema, a common response to inflammation or injury, indicates the body’s reparative efforts, while a heightened signal intensity on T2-weighted images typifies inflammation and is present in several conditions, including HADD (Figure 5).

Moreover, MRI occasionally displays the characteristic “arc and ring” pattern of the deposits, indicating a low signal intensity core of hydroxyapatite crystals encircled by a high signal intensity rim of inflammatory tissue [3]. This pattern serves as a valuable diagnostic marker in MRI readings of potential HADD patients.

**Figure 6:**
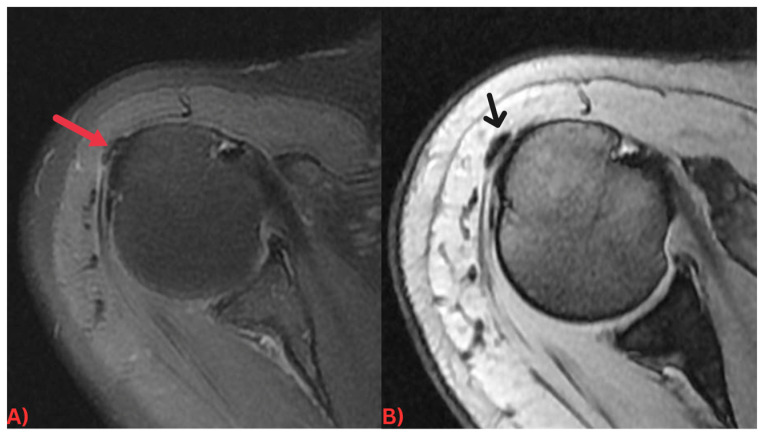
(**A**) Axial PD fat-saturated MRI image and (**B**) axial GRE sequence MRI of the right shoulder demonstrating a small well-defined low signal intensity calcification at the insertion of the supraspinatus tendon (red arrow). Note how the calcification is more visible and conspicuous on the GRE sequence (black arrow) due to a blooming artifact from the calcification.

Gradient recalled echo (GRE) sequences can reveal hydroxyapatite deposits as areas of signal voids or hypointensity due to the magnetic susceptibility effects of the calcified material. This is particularly useful in early-stage HADD, where the amount of hydroxyapatite deposition may be minimal and harder to detect with other imaging techniques (Figure 6).

#### 5.1.4. Ultrasound

Ultrasound utilizes high-frequency sound waves to generate images of the body’s internal structures. For HADD diagnosis, ultrasound is critical in identifying the size, shape, and location of hydroxyapatite deposits, which manifest as hyperechoic foci within soft tissues due to their high reflectivity [29].

**Figure 7:**
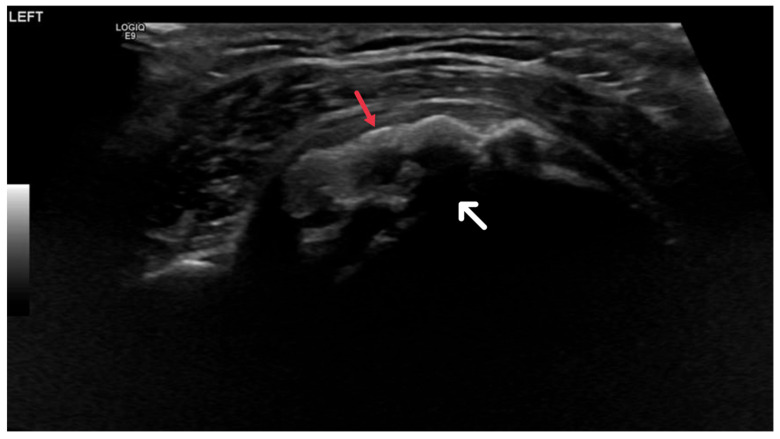
Ultrasound image showing calcific focus at the supraspinatus tendon insertion (red arrow) with posterior acoustic shadowing (white arrow).

These deposits may correspond to acoustic shadowing, a phenomenon where ultrasound waves are obstructed by the deposits, leading to an image shadow (Figure 7). This feature is of diagnostic value, as it differentiates hydroxyapatite deposits from other soft tissue abnormalities [30]. Furthermore, ultrasound can detect inflammation and tendon damage through increased echogenicity of the surrounding tissues and alterations in tendon size, shape, and echotexture [31].

Radiological findings of HADD are extensive and observable using various imaging modalities (Table 4). Combined with the patient’s clinical presentation and history, these findings aid in the accurate diagnosis of HADD and differentiation from other conditions [32]. 

## 6. HADD Can Mimic Other Diseases

HADD can mimic other diseases, such as tumoral calcinosis and aggressive neoplastic processes, presenting a diagnostic challenge.

Tumoral calcinosis, a rare condition, features large, calcified, periarticular soft tissue masses made of calcium phosphate crystals, often associated with hyperphosphatemia. Radiographs of tumoral calcinosis exhibit large, lobulated masses with a dense, amorphous calcification pattern. These can resemble the appearance of hydroxyapatite deposits in HADD, which similarly appear as amorphous, cloud-like densities in soft tissues [33].

Similarly, HADD’s bone erosion can mimic bone destruction in aggressive tumors. Particularly, malignant neoplastic processes can cause substantial bone destruction and cortical irregularities, mirroring those observed in advanced HADD cases. This resemblance complicates the differentiation of HADD from malignant tumors based solely on radiological findings [25,34].

However, several elements aid in distinguishing HADD from these conditions. Characteristic calcifications, usually located around joints and associated with bone erosion or cortical irregularities, are a unique feature of HADD [24]. In comparison, calcifications in tumoral calcinosis are typically larger and more lobulated, and bone destruction in aggressive neoplastic processes is more extensive and accompanies a soft tissue mass [33].

Additionally, the localization of calcification in HADD provides diagnostic clues. They are typically located in the tendons around joints, especially the shoulder, but can also be found in unusual locations like the hip, wrist, and spine. In contrast, calcifications in tumoral calcinosis are usually found in the periarticular soft tissues, and bone destruction in aggressive neoplastic processes can occur in any bone affected by the tumor [2].

Despite the challenge posed by radiological mimicry of other diseases, a comprehensive understanding of HADD’s imaging features aids in accurate diagnosis and suitable management. It is essential to consider the patient’s clinical history, physical examination findings, and laboratory results along with imaging findings for the correct diagnosis. As always, correlation with clinical findings is crucial in radiology, with HADD being no exception [8].

### 6.1. Differentiating HADD from Other Diseases Based on Imaging Findings

Differential diagnosis is a vital component of medical practices, providing doctors the tools to distinguish diseases with comparable symptomatology or manifestation. HADD introduces unique challenges to the differential diagnosis process, as it has a propensity to mimic other conditions. This mimicry is particularly apparent in radiological findings where HADD’s calcified deposits may appear to mirror diseases such as tumoral calcinosis or aggressive neoplastic processes. Thus, a comprehensive understanding of HADD’s imaging traits is imperative for an accurate diagnosis and suitable treatment management [3,25,33,34].

#### 6.1.1. The Acute Phase

Acute symptomatic HADD is often misdiagnosed as a traumatic or infectious process, especially when it affects the longus colli muscle at the cervical spine. In these scenarios, characteristic calcifications are key to differentiate HADD from infection. When individuals present with acute symptomatic HADD following minor trauma, there is a possibility that HADD’s calcifications may be misinterpreted as avulsion fragments. Nevertheless, avulsion fractures typically display a linear or incompletely corticated appearance, contrasting with HADD calcification’s more ambiguous or homogeneous display [35].

During HADD’s acute phase, patients might exhibit symptoms similar to those of traumatic injury or infection, including pain, swelling, and a reduced motion range in the affected area. In situations where HADD affects the longus colli muscle at the cervical spine, patients might also experience neck stiffness, odynophagia (painful swallowing), and dysphagia (difficulty swallowing). In rare instances, the swelling accompanying HADD in this region can lead to airway compromise [35].

The distinguishing factor between acute symptomatic HADD and a traumatic or infectious process is the characteristic calcifications seen in imaging studies. These calcifications are calcium apatite crystal deposits within and around connective tissues, a defining feature of HADD. In radiographic images, these calcifications often exhibit an amorphous, nebulous appearance, differing considerably from the linear or incompletely corticated appearance of avulsion fragments typically seen in traumatic injuries [35].

When minor trauma occurs, the misinterpretation of HADD’s calcifications as avulsion fragments might occur. Avulsion fractures result from a muscle’s forceful contraction or stretch, tearing away a bone fragment. These fractures are commonly associated with sports injuries and frequently observed in younger individuals engaged in activities involving sudden accelerations or decelerations. In imaging studies, avulsion fractures usually appear as linear or incompletely corticated bone fragments, contrasting sharply with the ambiguous or homogeneous display of HADD calcification [35].

Therefore, when symptoms suggestive of a traumatic or infectious process arise, especially in minor trauma scenarios, it is crucial to consider HADD as a potential differential diagnosis. A meticulous examination of imaging studies, focusing particularly on the presence and appearance of calcifications, can assist in distinguishing HADD from other conditions, guiding appropriate management [36].

#### 6.1.2. The Chronic Phase

Chronic symptomatic HADD frequently poses diagnostic challenges due to its similarity to other conditions like trauma’s late sequela, specifically heterotopic ossification or malignancy. Heterotopic ossification denotes the aberrant formation of true bone within extraskeletal soft tissues, which typically occurs post-trauma or surgery. Its imaging characteristics can mimic HADD, but the key differentiating factor is a corticated margin’s presence surrounding the heterotopic ossification, which is absent in HADD [35].

One of the challenges encountered during the process of distinguishing HADD from other diseases is the presence of bony erosion and periosteal reaction that could be mistaken for signs of a soft tissue malignancy or sarcoma [35]. These types of cancers originate in soft tissues such as fat, muscle, nerves, fibrous tissues, blood vessels, or deep-skin tissues, making the differentiation complex due to similar calcification and bone erosion patterns on imaging studies. Nevertheless, an absence of a discrete soft tissue mass, alongside calcification located at a typical HADD site, can support the differentiation between HADD and a neoplastic process.

Concluding the diagnostic perspective, the differential diagnosis of HADD relies heavily on the recognition of characteristic imaging features and an understanding of how these differ from those of other diseases [3]. This ability to distinguish HADD from other conditions based on imaging findings underpins the precision of diagnosis and tailoring of the most suitable management approach.

#### 6.1.3. Other Conditions Acting as Differentials in HADD

##### Trauma

Trauma, either acute or chronic, is a significant factor that may lead to calcification within the affected soft tissues. This calcification can be easily confused with HADD. In cases of trauma, be it a singular traumatic event or an ongoing history of injury, radiological findings often reveal distinctive signs such as fracture lines, bone contusions, or other evidence that is entirely distinct from the amorphous calcifications observed in HADD. The differentiation process is vital, as it relies on a combination of the patient’s history, clinical examinations, and diverse imaging modalities like X-rays, CT scans, or MRI [35]. It is worth emphasizing the significance of recognizing the exact location and pattern of calcification and the absence of systemic symptoms typically linked to HADD, as this understanding can be instrumental in determining the correct treatment approach, which significantly differs from HADD management.

##### Infection

Chronic infections, particularly those that infiltrate the joints or surrounding tissues, often present a clinical picture that might lead to calcifications resembling HADD. The nature of these calcifications could be bacterial, fungal, or viral, usually localized or generalized within the affected region. The importance of distinguishing infection from HADD cannot be overstated, as it is a matter of employing entirely different therapeutic strategies. This approach to diagnosis involves a detailed clinical history, laboratory markers for infection (such as elevated white blood cell counts, C-reactive protein, or culture results), and relevant imaging techniques [35]. Contrary to HADD, infections often necessitate specific treatments like antimicrobial therapy or surgical interventions, thus underlining the vital importance of accurate differentiation.

##### Pseudohypoparathyroidism

Pseudohypoparathyroidism (PHP) is characterized by features like short stature, obesity, round face, and intellectual disability. It may also cause calcifications within soft tissues that resemble HADD [14]. However, the diagnosis process involves the observation of unique laboratory findings, such as low calcium and high phosphate levels and specific skeletal abnormalities on radiological examinations such as shortening of the fourth metacarpal. Further assessment may include a detailed family history and identifying additional features related to Albright’s hereditary osteodystrophy, making the management of PHP remarkably different from HADD.

##### Hyperparathyroidism

Hyperparathyroidism can also induce generalized calcifications within soft tissues, creating confusion with HADD. The hallmarks of this disorder include increased levels of PTH, leading to disturbances in calcium and phosphate metabolism. Differentiating this condition from HADD relies on laboratory findings, such as elevated serum calcium and PTH levels, and radiological imaging that might reveal calcification in various parts of the body [15]. Proper differentiation from HADD is crucial, as its management might necessitate measures, like medication, dietary modification, or even surgical intervention, that are tailored to the underlying cause.

##### CPPD (Chondrocalcinosis)

Calcium pyrophosphate deposition disease (CPPD) sets itself apart from HADD due to its more defined calcification patterns. Unlike HADD’s typical amorphous calcifications, CPPD manifests in acute or chronic joint pain and is often linked with underlying metabolic disorders. Specific radiographic findings and the involvement of certain joints, predominantly affecting areas like the knees or wrists, further support its differentiation from HADD. The treatment approach for CPPD, encompassing NSAIDs, corticosteroids, or colchicine, varies markedly from that for HADD.

In summary, the complexity of conditions like trauma, infections, PHP, hyperparathyroidism, and CPPD necessitates a nuanced understanding to differentiate them from HADD. Through careful examination, clinical expertise, and the use of appropriate diagnostic tools, the proper treatment and management approach for each condition can be effectively implemented.

## 7. Treatment of HADD

In the treatment of HADD, a myriad of strategies have been utilized, combining conservative and interventional approaches [37]. These methods are employed considering the disease’s severity, the deposit location, and the patient’s overall health status.

The first line of defense in managing HADD often consists of conservative treatment approaches. These include rest, physical therapy, and administration of non-steroidal anti-inflammatory drugs (NSAIDs) [38]. The importance of rest as a fundamental component of this approach cannot be overstated, providing the body an opportunity to heal and recover naturally, thus reducing HADD-induced inflammation and pain [37].

Physical therapy, a significant element in HADD management, works towards improving joint function, increasing range of motion, and strengthening muscles surrounding the affected joint [39]. NSAIDs, often employed to reduce inflammation and alleviate pain, function by inhibiting the production of certain inflammation-causing chemicals in the body [40].

In instances where conservative treatment strategies fail to offer sufficient relief, interventional treatments are considered. These include extracorporeal shock wave therapy (ESWT) and surgical intervention [41]. ESWT, a non-invasive procedure, uses sound waves to break up hydroxyapatite deposits, facilitating their resorption. Particularly effective in cases where deposits are near the skin surface, this therapy represents an innovative approach to treatment [42,43].

Surgical intervention, reserved typically for severe cases, involves arthroscopic or open surgery to remove deposits [44,45]. The decision to proceed with surgery requires careful evaluation of the patient’s overall health status, the severity of their symptoms, and their response to conservative treatment. It is of paramount importance that patients work closely with their healthcare provider in developing a treatment plan tailored to their specific needs and circumstances [46].

### Role of the Imaging Specialist in the Treatment of HADD

The role of the imaging specialist in the treatment of HADD is indeed crucial. Imaging specialists play a key role in the diagnosis of HADD, as the disease is primarily identified through imaging studies. They are responsible for identifying the hydroxyapatite deposits and differentiating HADD from other conditions that may present with similar imaging findings [1,47].

The imaging specialist’s role begins with the initial diagnosis. Using imaging modalities such as X-ray, CT, MRI, and ultrasound, imaging specialists can identify the characteristic calcifications associated with HADD. These deposits typically appear as areas of increased density or signal in these imaging studies, allowing the imaging specialist to differentiate HADD from other conditions that may present with similar symptoms but different imaging findings [47].

In addition to identifying the hydroxyapatite deposits, imaging specialists also play a crucial role in assessing the extent of the disease. They can determine the size and location of the deposits as well as any associated soft tissue changes or bone erosion. This information is vital for planning the appropriate treatment strategy [47].

**Figure 8:**
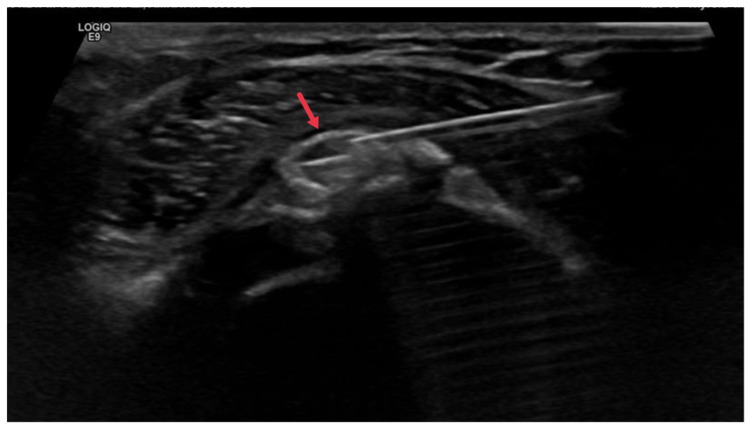
Ultrasound image showing a percutaneous calcific lavage procedure with a needle within the calcific focus during the irrigation (red arrow).

Imaging specialists also play a significant role in the treatment of HADD through image-guided procedures. For instance, they may perform image-guided needle aspiration or lavage to remove the deposits. This procedure involves using imaging guidance to insert a needle into the affected area and aspirate the calcific deposits (Figure 8). This can help to alleviate symptoms and promote healing [48].

In addition, imaging specialists may guide the placement of corticosteroid injections to reduce inflammation. These injections can be administered directly into the bursae or soft tissues surrounding the affected joint, providing immediate relief from pain and inflammation. The precise placement of these injections is crucial to ensure their effectiveness and minimize potential side effects, and the expertise of the imaging specialist is invaluable [41].

Furthermore, imaging specialists play a key role in monitoring the progress of the disease and the effectiveness of treatment. Through follow-up imaging studies, they can assess whether the hydroxyapatite deposits are decreasing in size or disappearing altogether, indicating a positive response to treatment. They can also identify any potential complications or new areas of calcification [47].

Therefore, the role of the imaging specialist in the treatment of HADD extends beyond the initial diagnosis. They are integral to the treatment and management of the disease, providing valuable insights through their expertise in imaging studies and image-guided procedures. Their contributions are essential to ensuring the most effective treatment strategy for each individual patient, ultimately leading to better patient outcomes [1,47].

Imaging specialists are integral to multidisciplinary approaches to managing HADD. Collaborating with healthcare professionals like rheumatologists, orthopedic surgeons, and physical therapists, they provide key imaging findings that guide treatment strategies. For example, the extent of hydroxyapatite deposition can inform decisions about surgical intervention, while signs of inflammation can help tailor therapeutic interventions. Additionally, imaging specialists aid in patient education, explaining imaging results to help patients better understand their condition and fostering improved treatment adherence and outcomes.

## 8. Future Recommendations

Hydroxyapatite deposition disease (HADD) is a convoluted medical condition, with significant advances in its comprehension and administration observed over the years. Yet, several areas remain unexplored and necessitate further scientific scrutiny and inquiry.

A prime area that calls for an intensified probe lies in the pathogenesis of HADD. The deposition of hydroxyapatite crystals in soft tissues, a pivotal phenomenon in HADD, still leaves researchers speculating about its precise mechanisms. Although four potential pathways have been proposed, their exact implications remain elusive. Therefore, it is crucial to shed light on these mechanisms. Doing so could pave the way for innovative therapeutic strategies that prevent or reverse these processes. Expounding the interactions between genetic, environmental, and cellular factors in HADD pathogenesis could further elucidate this intricate disease process.

Simultaneously, the role of imaging specialists in the diagnostic and management process of HADD is another area that requires further exploration. Recent advancements in artificial intelligence (AI) can be a potential game-changer in this respect. AI promises transformative tools capable of enhancing image analysis, boosting diagnostic precision, and even offering prognostic capabilities for disease progression [49]. Consequently, future studies exploring AI applications in HADD diagnosis and treatment could be pivotal. For instance, machine learning algorithms can be designed to recognize HADD’s imaging features, distinguish it from other conditions, and even predict the outcomes of treatment approaches [50].

Furthermore, the scope of imaging specialists’ roles could transcend diagnosis and treatment. As subject matter experts in HADD imaging findings, imaging specialists are strategically positioned to educate patients about their condition, demystify imaging results, and promote apt treatment strategies, thus evolving into patient advocates.

## 9. Conclusions

In conclusion, HADD’s complexity is manifested in its diagnostic and treatment challenges. It can present itself in various bodily locations and imitate other conditions, necessitating a profound understanding of its radiological findings for precise diagnosis. A blend of conservative and interventionist approaches formulates the treatment plan, which is tailored to the disease severity and the overall health status of the patient. The pivotal role of radiologists in diagnosing and treating HADD underscores the importance of a multidisciplinary approach to managing this disease. Notwithstanding the substantial strides made in HADD comprehension and management, several areas still demand intensified research. Pioneering research in these areas, especially in demystifying HADD pathogenesis and AI’s role in radiology, could potentially usher in an era of enhanced diagnostic tools and treatment strategies. This would significantly boost outcomes for patients grappling with HADD.

## Figures and Tables

**Table 1:** Pathogenic pathways of HADD.

Pathogenic Pathway	Key Players	Outcome
Degenerative calcification	Tendon, vascular ischemia, trauma	Deposition of calcified material
Reactive or cell-mediated calcification	Chondrocytes, phagocytosing cells	Calcium deposition and resorption of calcified material
Process similar to endochondral ossification	Cartilage, bone	Abnormal calcification within soft tissues
Erroneous differentiation of tendon-derived stem cells	Tendon-derived stem cells, chondrocytes, osteoblasts	Development of HADD due to calcium deposition

**Table 2:** Predisposing risk factors for HADD.

Risk Factor	Type	Possible Mechanism
Diabetes	Metabolic	Increased oxidative stress and chronic low-grade inflammation
Thyroid disorders	Metabolic	Disruptions in metabolic regulation
Estrogen metabolism disorders	Metabolic	Imbalances in hormones affecting bone health
HLA-A1 genotype	Genetic	Potential immunological component

**Table 3:** Stages of HADD.

Stage	Description	Symptoms	Radiographic Findings
Precalcific	Initial phase characterized by impaired perfusion and resultant focal hypoxia, triggering fibrocartilaginous transformation	Often asymptomatic	Not typically visible on radiographs
Calcific	Marked by the deposition of calcium crystals; further subdivided into formative, resting, and resorptive phases	Acute pain during resorptive phase	Visible calcifications, especially during the resorptive phase
Postcalcific	Occurs when the void from the resorptive phase of the calcific stage is replaced by granulation and scar tissue	Resolution of disease	Healing process visible, calcifications typically absent

**Table 4:** Radiological findings in HADD.

Imaging Modality	Appearance of Hydroxyapatite Deposits	Associated Findings
Plain radiographs	Amorphous, cloud-like densities	Bone erosion or cortical irregularities
Computed tomography (CT)	High-density areas	Surrounding inflammation
Magnetic resonance imaging (MRI)	Low signal intensity areas	Surrounding inflammation, “arc and ring” pattern
Ultrasound	Hyperechoic foci	Acoustic shadowing, inflammation, tendon damage

## Data Availability

Not applicable.
